# Patterns of use and adverse events reported among persons who regularly inject buprenorphine: a systematic review

**DOI:** 10.1186/s12954-022-00695-5

**Published:** 2022-10-13

**Authors:** Nikki Bozinoff, Vitor Soares Tardelli, Dafna Sara Rubin-Kahana, Bernard Le Foll

**Affiliations:** 1 Campbell Family Mental Health Research Institute, Centre for Addiction and Mental Health, ON Toronto, Canada; 2 Department of Family and Community Medicine, University of Toronto, Toronto, ON Canada; 3 Addictions Division, Centre for Addiction and Mental Health, Toronto, ON Canada; 4 Departamento de Psiquiatria, Universidade Federal de Sao Paulo, São Paulo, Brazil; 5 Translational Addiction Research Laboratory, Centre for Addiction and Mental Health, Toronto, ON Canada; 6 Department of Psychiatry, University of Toronto, Toronto, ON Canada; 7 Child, Youth, and Family Services, Centre for Addiction and Mental Health, Toronto, ON Canada; 8 Division of Neurosciences and Clinical Translation, Department of Psychiatry, University of Toronto, Toronto, ON Canada; 9 Department of Pharmacology and Toxicology, University of Toronto, Toronto, ON Canada; 10 Waypoint Research Institute, Waypoint Centre for Mental Health Care, Penetanguishene, ON Canada

**Keywords:** Buprenorphine, Opioid use disorder, Opioids, iOAT, Overdose, Intravenous, Misuse, Abuse

## Abstract

**Background and Aims:**

Given the ongoing opioid crisis, novel interventions to treat severe opioid use disorder (OUD) are urgently needed. Injectable opioid agonist therapy (iOAT) with diacetylmorphine or hydromorphone is effective for the treatment of severe, treatment-refractory OUD, however barriers to implementation persist. Intravenous buprenorphine for the treatment of OUD (BUP iOAT) has several possible advantages over traditional iOAT, including a safety profile that might enable take-home dosing. We aimed to characterize injecting practices among real-world populations of persons who regularly inject buprenorphine, as well as associated adverse events reported in order to inform a possible future BUP iOAT intervention.

**Methods:**

We conducted a systematic review. We searched MEDLINE, EMBASE, and PsycINFO from inception through July 2020 and used backwards citation screening to search for publications reporting on dose, frequency among persons who regularly inject the drug, or adverse events associated with intravenous use of buprenorphine. The review was limited to English language publications and there was no limitation on study type. Study quality and risk of bias was assessed using the Mixed Methods Appraisal Tool. Narrative synthesis was used in reporting the results.

**Results:**

Eighty-eight studies were included in our review. Regular injection of buprenorphine was identified across diverse settings world-wide. Daily dose of oral buprenorphine injected was < 1–12 mg. Frequency of injection was 0–10 times daily. Adverse events could be characterized as known side effects of opioids/buprenorphine or injection-related complications. Most studies were deemed to be of low quality.

**Conclusions:**

Extramedical, intravenous use of buprenorphine, continues to be documented. BUP iOAT may be feasible and results may inform the development of a study to test the efficacy and safety of such an intervention. Future work should also examine acceptability among people with severe OUD in North America. Our review was limited by the quality of included studies.

**Supplementary Information:**

The online version contains supplementary material available at 10.1186/s12954-022-00695-5.

## Introduction

The opioid overdose crisis continues unabated in the USA and Canada. In 2020, over 6300 people died in Canada and over 90,000 died in the USA of opioid-related overdose [1, 2]. Reports across multiple jurisdictions have confirmed that the COVID-19 pandemic has exacerbated the crisis [3–5].

Injectable diacetylmorphine has been used in the UK and Europe for decades [6, 7], and is effective for the treatment of severe OUD, not responsive to oral opioid agonist therapy (OAT) [6, 8–11]. Injectable hydromorphone has also emerged as a novel therapy for OUD following the publication of a randomized controlled trial that demonstrated non-inferiority compared with diacetylmorphine for severe, treatment-refractory OUD [9]. Benefits of injectable OAT (iOAT) in this population include improved retention in treatment compared with oral methadone alone, and reduction in the use of non-prescribed opioids [9, 10]. Multiple studies have also demonstrated iOAT to be cost-effective for severe OUD [12–15].

Despite the urgent need for treatment options in the setting of a toxic drug supply and mounting overdose deaths across the North America, the widespread implementation of iOAT has not taken place [16]. A recent environmental scan of iOAT programs across Canada revealed only 14 programs with total capacity for 420 clients. Barriers to the scale up of iOAT identified included, the high cost of infrastructure and personnel required to operate a program that directly supervises people who inject multiple times daily, and lack of government funding for high-dose liquid hydromorphone or diacetylmorphine in multiple provinces [16].

Given the ongoing opioid crisis, there remains a need for novel treatment options for persons not benefitting from oral OAT. Buprenorphine is a partial mu-opioid receptor agonist which is indicated as a first-line treatment for OUD owing to its favorable safety profile—it carries a much lower risk of respiratory depression and overdose when compared to full opioid agonists [17]. In Canada, buprenorphine is available for the treatment of OUD as a sublingual tablet co-formulated with naloxone (hereinafter BNX), and as a buccal film. As a result of its safety profile, it is feasible and non-inferior to methadone to provide BNX with a large number of take-home doses [18]. Two long acting formulations are also available: extended-release buprenorphine for subcutaneous injection by a medical provider at 4 week intervals, and buprenorphine subdermal implants lasting 6 months in duration [19]. Transdermal buprenorphine patches are also available however, are only approved in the treatment of pain.

Interestingly, several preclinical [20–22] and clinical studies [23–25] have shown that buprenorphine can produce reinforcing and rewarding effects under appropriate conditions. Specifically, where intravenous buprenorphine was administered to detoxified persons with opioid use disorder, participants reported euphoria, liking the drug’s effects, and a desire to continue taking it [23–25]. In countries in which buprenorphine is widely available, cohorts of people who use intravenous buprenorphine as a drug of choice have been described [26–29]. In fact, it was concerns regarding early reports of extramedical use of buprenorphine [30, 31] that led to the creation of BNX [22, 32], the “abuse-deterrent” formulation most commonly used in the USA and Canada. Despite the widespread use of this “abuse-deterrent” formulation, regular intravenous use of BNX has been well-described [33–35].

Injectable buprenorphine as an alternative to injectable diacetylmorphine or hydromorphone has a number of possible benefits. Most significantly, BUP iOAT could be a safer form of iOAT owing to lower risk of respiratory depression and overdose [36], could potentially be disseminated in low barrier settings (e.g. take-home doses), may be associated with reduced stigma, and may facilitate transition to traditional oral OAT. In France, a recent cross-sectional survey among people with OUD not responsive to oral treatments indicated a strong willingness to consider treatment with BUP iOAT were it available (83% of respondents) [37].

Nevertheless, no clinical trials on the use of BUP iOAT as a novel iOAT exist, it is unclear what dose and frequency of injection would be required to retain people in treatment, and adverse events related to injection of this medication are important to understand. Given the urgency of the opioid crisis, and the need for novel therapeutic options for people with severe refractory OUD, we undertook a systematic review to characterize injecting practices among real-world persons who regularly inject buprenorphine, as well as associated adverse events reported.

## Methods

We adhered to the Preferred Reporting Items for Systematic Reviews and Meta-Analyses (PRISMA) 2020 guideline in conducting and reporting this systematic review [38]. The protocol was registered on PROSPERO [39]. We searched the following electronic bibliographic databases from inception: MEDLINE, EMBASE, PsycINFO and also hand searched the reference lists of included studies from the initial search. We searched all available record fields using natural language search terms capturing three conceptual areas relevant to our search: (1) “Buprenorphine” (2) “injection” and (3) “misuse” (see Additional file 1: Appendix 1 for full search strategy). The initial search was conducted in July 2020.

Titles and abstracts of studies retrieved using the search strategy above were screened independently by two review authors (NB and DRK) to identify studies that potentially met the inclusion criteria. The full text of these potentially eligible studies was retrieved and independently assessed for eligibility by two team members (NB and VST). Any disagreements at screening were resolved through discussion with a third member of the study team. Studies were included if they reported on the dose or frequency of intravenous buprenorphine use among real-world populations of persons with opioid use disorder who regularly inject buprenorphine, or if they reported on adverse events associated with intravenous use of buprenorphine. There was no restriction on study type; however lab-based studies and studies related to the use of buprenorphine in the management of pain were excluded. Studies related to extended-release formulations of buprenorphine where subcutaneous administration is appropriate were excluded. Owing to resource limitations, only English language publications were included. Data was managed in Covidence systematic review software (2021), Veritas Health Innovation, Melbourne, Australia.

A standardized data abstraction form was developed and used to extract data from the included studies for evidence synthesis. Extracted information included: bibliographical information, study setting, study population, year of data collection, details about the outcomes (including dose, frequency, formulation of buprenorphine used and adverse event(s) reported). Descriptions of adverse events were taken verbatim from the text and no attempt to verify causality was made. Two reviewers extracted data independently and discrepancies were resolved through discussion (with a third author where necessary). For studies reporting on either dose or frequency of use, where one element was missing, we attempted to request this data from the authors via email. Where information remained missing, it was left blank in the table.

Study quality and risk of bias was assessed using the Mixed Methods Appraisal Tool (MMAT), which provides a set of criteria for appraising methodological quality of quantitative, qualitative and mixed methods studies [40, 41]. Quality scores were calculated independently by two reviewers using the MMAT tool. For mixed methods studies, we used the lowest score from amongst the study components. Any conflicts were resolved by a third reviewer. Scores of ≤ 3 were considered to be of low quality and at high risk of bias [42].

Because we anticipated significant heterogeneity in the way results were reported, data across studies were summarized using narrative synthesis. We adhered to the guidance on narrative synthesis in systematic reviews developed by Popay et al., (2006), which provides guidance on maintaining transparency, trustworthiness and avoiding bias in the composite of findings [43].

## Results

**Fig. 1:**
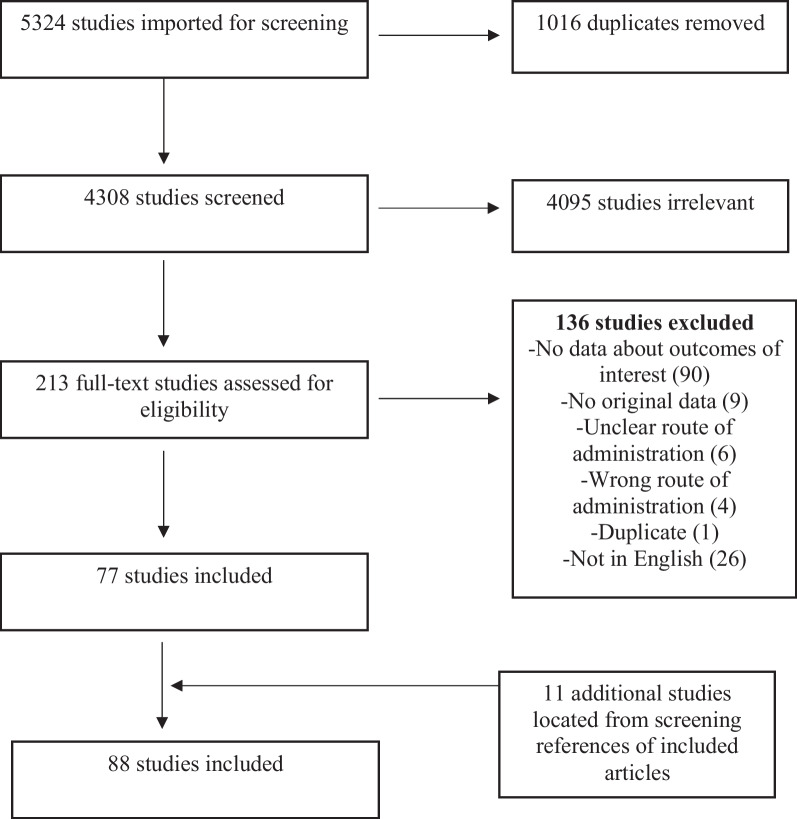
PRISMA flow diagram

Figure 1 describes the search and selection process using the PRISMA flow diagram. Five thousand, three-hundred and twenty-four studies were found in the search and imported for screening. After duplicates were removed, 4308 studies were included in first stage screening and titles and abstracts were reviewed. Two-hundred and thirteen studies passed first-stage screening and full texts were assessed for inclusion. After second stage screening, 77 studies were included and 11 additional studies were included after reviewing the reference lists of included studies for a total of 88 included studies. The included studies were published between 1984 and 2020 and were from multiple cities across Australia, Bangladesh, China, Finland, France, Georgia, India, Iran, Malaysia, Nepal, New Zealand, Scotland, Singapore, Spain, Turkey, and the USA (Table 1, 2).

**Table 1:** Studies discussing dose and frequency of intravenous buprenorphine use

Study citation	Location	Study population	Study design	Year data was collected	Buprenorphine formulation injected	Dose/frequency of use reported	Quality Assessment [1–5]
Aalto [52]	Kotka, Finland	27 people who use IV buprenorphine	Quantitative descriptive	2004–2005		Dose: 8.1 mg/day	2
Ahmadi^a,%^ [67]	Shiraz, Iran	204 males who use IV buprenorphine	Clinical trial	2002	Buprenorphine ampoules	Dose: mean = 3.86 amps/day (SD = 2.61), range 1–19 amps/day. *1 amp contains 0.3 mg of buprenorphine in 1 ml, therefore a mean daily dose of 1.16 mg/day (SD = 0.78), range 0.3–5.7 mg/day	3
Ahmadi^b,%^ [26]	Shiraz, Iran	204 males who use IV buprenorphine	Clinical trial	2002	Buprenorphine ampoules	Dose: mean = 3.7 amps/day (SD = 2.6), range 1–19 amps/day. *1 amp contains 0.3 mg of buprenorphine in 1 ml, therefore a mean daily dose of 1.1 mg/day (SD = 0.78), range 0.3–5.7 mg/day	3
Ahmadi^c^ [68]	Shiraz, Iran	108 males who use IV buprenorphine	Clinical trial	2002	Buprenorphine ampoules	Dose: mean = 4.6 amps/day (SD = 3.1), range 1–17 amps/day. *1 amp contains 0.3 mg of buprenorphine in 1 ml, therefore a mean daily dose of 1.38 mg/day (SD = 0.93), range 0.3–5.1 mg/day	3
Ahmed [51]	Dhaka, Bangladesh	30 males with extramedical use of buprenorphine	Quantitative descriptive	1995	Buprenorphine ampoules	Dose: Range < 1–10 amps/day, 60% used 2–5 amps per day. *1 amp contains 0.6 mg of buprenorphine in 2 ml therefore range of < 0.6–6 mg/day, 60% used 1.2–3 mg/day; Frequency range 1–10 times/day, 86.7% used 2–5 times daily	2
Aich [69]	Bhairahawa, Nepal	76 people with OUD	Quantitative descriptive	2003–2004	Buprenorphine ampoules	Dose: 0.6 mg/injection; Frequency: 2–4 times/day	4
Aitken [27]	Melbourne, Australia	316 PWID	Quantitative descriptive	2005–2006	SL buprenorphine (spitbacks)	Dose: 2-12 mg/day; Frequency: 10.4–10.5 times /week	3
Alho [70]	Helsinki, Finland	176 people needle exchange clients	Quantitative descriptive	2005	SL buprenorphine and BNX	Dose: 7 mg/day; Frequency: 81.8% daily users (41.6% 3–4 injections/day)	3
Ambekar [28]	Multiple sites in India	902 male PWIDs at harm reduction centres	Quantitative descriptive		Buprenorphine ampoules	Frequency: 48.9% daily injectors; 66.2% more than one injection/day on using days	3
Basu [71]	Chandigarh, India	3 males (30, 26, 25 years) who use IV buprenorphine	Case series		Buprenorphine ampoules	1st case: 0.3 mg 2–3 times per day, escalating to 2–4 times that dose; 2nd case: 1.8 mg daily; case 3: 2.4 mg daily	1
Basu [72]	Chandigarh, India	94 males with extramedical use of buprenorphine	Quantitative descriptive	1987–1993	Buprenorphine ampoules	Dose: 1.8 mg/day	2
Bruce [73]	Kuala Lumpur, Malaysia	19 males who use IV buprenorphine	Case series	2006	SL buprenorphine	Dose: 1-4 mg/injection; Frequency: 2–4 times/day	2
Bruce [35]	Kuala Lumpur, Malaysia	41 people who use IV buprenorphine	Quantitative descriptive	2007	SL buprenorphine and BNX	Dose: 1.9 mg/day before BNX, 2.5 mg/day with BNX; Frequency: 3.9–4.3 injections/day	3
Chowdhury [74]	Guwahati, India	38 year old male	Case study		SL buprenorphine	Dose: 0.8–1.6 mg/day	1
Feeney [75]	Brisbane, Australia	24 year old female	Case study		SL buprenorphine	Dose: 8 mg/injection	1
Horyniak [76]	Melbourne, Australia	23 people who use IV buprenorphine	Mixed methods	2006	SL buprenorphine and BNX (mixed with saliva or lemon juice)	Frequency: up to four times/day among daily injectors	4
Kulaksızoglu [77]	Antalya, Turkey	19 year old male	Case study	2018	SL BNX (dissolved in hot water)	Dose: 10 mg/day	1
Kumar [50]	Madras, India	100 PWIDs	Quantitative descriptive	1998	SL buprenorphine	Frequency: 92.8% 1–3 times/day; 7.1% more than 4 times/day	4
Lavelle [78]	Glasgow, Scotland	78 clients from residential drug treatment centres	Quantitative descriptive	1989–1990	SL buprenorphine	Dose: 1.5 mg/day; Frequency: 243 using days/year; 58% 5–7 days/week	3
Lee [79]		mid-40 s female	Case study		SL buprenorphine (dissolved in hot water)	Dose: 1–3 tablets/injection	1
Liu [80]	Multiple sites in China	1235 people with OUD and a history of buprenorphine use for at least three days	Quantitative descriptive	2000–2001	SL buprenorphine	Dose: 0.5–0.8 mg/injection; Frequency: 2.0–2.8 times/day	4
Nizamie (1990)	India	32 year old male	Case study	1988	Buprenorphine ampoules	Dose: 2–4 amps/day	1
Ng [81]	Singapore, Singapore	120 people who use IV buprenorphine	Quantitative descriptive	2005–2006	SL buprenorphine	Dose: 7.4–9.0 mg/day	3
Obadia [82]	Marseille, France	343 PWIDs	Quantitative descriptive	1997	SL buprenorphine	Frequency: 60.2–76.8% inject once a day or more	5
Otiashvili [83]	Multiples sites in Georgia	381 PWIDs	Quantitative descriptive	2007	SL buprenorphine	Dose: 1-8 mg/injection (44% injected 1 mg, 45.8% 2 mg, 9% 4 mg, 0.56% 8 mg)	3
Peyrière [60]		33 year old male and 50 year old male	Case series	2007	SL buprenorphine	Dose: 4 mg/injection; frequency: 3–5 times/day	2
Piralishvili [84]	Tbilisi, Georgia	80 people who use IV buprenorphine	Clinical trial	2011	SL BNX	Dose: 1.75 mg/day; Frequency: 15.2 days in the past 30 days	4
Quigley [31]	West Perth, Australia	24 year old male who uses IV buprenorphine	Case report	1983	Buprenorphine ampoules	Dose: 4.5 mg/day	1
Robinson [34]	Wellington, New Zealand	2 consecutive surveys (54 and 44 people respectively) presenting for OUD treatment	Quantitative descriptive	1990–1992	SL buprenorphine and BNX	Dose: 0.6 mg of buprenorphine/injection on first survey, 0.4/0.34 mg of BNX/injection on second survey	3
Roux [47]	Multiple sites in France	111 clients receiving OAT with buprenorphine	Quantitative descriptive	2004–2005	SL buprenorphine	Frequency: 5% reported at least daily injection	5
Roux [57]	Multiple sites in France	371 PWID with OUD	Quantitative descriptive	2015	SL buprenorphine	Dose: median of 12 mg/day; Frequency: median of 3 injections/day (IQR: 2–4)	4
San [85]	Barcelona, Spain	188 (1988) and 197 (1990) heroin-dependent individuals	Quantitative descriptive	1988 and 1990	SL buprenorphine	Dose: 0.6–0.8 mg/day (1990) and 1.4–4.1 mg/day (1988)	5
Singh [86]	India	24 year old male	Case study		Buprenorphine ampoules	Dose: 24 mg/day, later 2.4–3.6 mg/day; Frequency: 5–6 injections/day	1
Singh [87]	Chandigarh, India	18 people with extramedical buprenorphine use	Case series	1987–1990	Buprenorphine ampoules	Dose: mean = 3 mg/day, range = 1-7 mg/day Frequency: 3–4 injections/day	3
Torrens [88]	Barcelona, Spain	22 buprenorphine and 45 heroin-dependent individuals	Quantitative descriptive	1988–1989	SL buprenorphine	Dose: 1.9 mg/day; Frequency: 3–4 times/day	4
Valenciano [89]	Multiple sites in France	1004 clients at syringe-exchange programs	Quantitative descriptive	1998	SL buprenorphine	Frequency: 1 injection/day	4
Vicknasingam [90]	Multiple sites in Malaysia	276 people who use IV buprenorphine; for the second wave 77/276 were re-interviewed 77/276 and additional 171 new participants included	Mixed methods	2006–2007	SL buprenorphine for first survey, BNX in second survey	Dose: first wave—96% used up to 2 mg/injection, second wave 81% used up to 2 mg/using day; Frequency: first wave—63% reported at least daily use, second wave—34% reported at least daily use	3
White [91]	Multiple sites in Australia	16 people who inject BNX films	Qualitative	2012–2013	Buccal BNX (films; spitbacks)	Frequency: 37.5% used daily, of which 83.4% (31.2% of the total) used > 5 times/day	5
Winslow [92]	Singapore, Singapore	120 people with extramedical use of buprenorphine, enrolled in treatment	Quantitative descriptive	2005–2006	SL buprenorphine	Dose: 7.7 (SD 4.8) mg/day	3
Winslow [93]	Singapore, Singapore	106 PWIDs presenting to an addictions management programme	Quantitative descriptive	2005–2006	SL buprenorphine	Frequency: "many" injected 3–4 times/day	5
Winstock [94]	Multiple sites in Australia	442 clients receiving methadone and 66 receiving supervised buprenorphine at community pharmacies	Quantitative descriptive	2005	SL buprenorphine	Dose: median amount injected on last injection was 6 mg (mean = 5.8; SD = 3.1; range = 2–10 mg)	4
Yeo [95]	Singapore, Singapore	8 clients aged 26–46	Case series	2005	SL buprenorphine (tablets dissolved in hot water)	Dose: 1-2 mg/injection (one of the cases)	2

*BNX* buprenorphine/naloxone, *IV* intravenous, *IQR* Interquartile range, *OAT* opioid agonist therapy, *OUD* opioid use disorder, *PWID* people who inject drugs, *SD* standard deviation, *SL* sublingual

% = these two studies report on the same study population with outcome measures at different time points. Dose and frequency of IV buprenorphine use was measured at baseline in both cases

**Table 2:** Studies discussing adverse events associated with intravenous buprenorphine use

Study citation	Location	Study population	Study design	Year data was collected	Buprenorphine formulation injected	Adverse event(s)	Quality Assessment [1–5]
Aboltins [96]	Melbourne, Australia	28 year old female	Case study		SL buprenorphine (spitbacks)	Fungal endophtalmitis	1
Aich [69]	Bhairahawa, Nepal	76 people with OUD	Quantitative descriptive	2003–2004	Buprenorphine ampoules	Thrombophlebitis, HIV, cellulitis, abscess	4
Alvarez [97]	Barcelona, Spain	32 year old male	Case study		SL buprenorphine	S. marcescens endopthalmitis resulting in blindness	1
Ambekar [28]	Multiple sites in India	902 male PWIDs at harm reduction centres	Quantitative descriptive		Buprenorphine ampoules	Abscess, blocked veins, overdose	3
Berson [61]	Clichy, France	33 year old male, 27 year old male, 28 year old male, 31 year old male	Case series	1996–1998	SL buprenorphine	Acute hepatitis in the context of chronic HCV	2
Boggs [98]	USA	29 year old female and 30 year old female	Case series		SL buprenorphine/BNX	Anaphylaxis and death	1
Bouquie [99]	Nantes, France	33 year old male	Case study	2014	SL buprenorphine	Livedo-like dermatitis with necrotic lesion	1
Boyd [100]	Helsinki, Finland	Records of 308 people with opioid overdose	Case series	1996–2002	SL buprenorphine	Overdose	3
Bruce [35]	Kuala Lumpur, Malaysia	41 people who use IV buprenorphine	Quantitative descriptive	2007	SL buprenorphine/BNX	Opioid withdrawal	3
Cassoux [101]	France	22 year old male, 25 year old male, 27 year old male, 30 year old male	Case series		SL buprenorphine (spitbacks, dissolved tabs in lemon juice)	Ocular candidiasis, septicemia, skin abscess, cervical lymphadenopathy, scalp nodules, wrist arthritis, folliculitis, chorioretinal lesion	2
Chai [102]	Singapore, Singapore	92 hospitalized patients	Case series	2003–2005	SL buprenorphine	Bacteremia, endocarditis, septic pulmonary emboli	2
Chew [103]	Singapore, Singapore	30 F, 35 M, 40 M, 60 M	Case series	2006	SL buprenorphine	Deep venous thrombosis, hand ischemia (thrombosis of brachial artery); epidural, limbs, and popliteal fossa abscesses	2
Chong [104]	Singapore, Singapore	12 clients aged 22–49	Case series	2005–2006	SL buprenorphine	Endocarditis, pneumonia, abscesses, septic shock, disseminated intravascular coagulation, acute heart and renal failure, among others	2
Chowdhury [74]	Guwahati, India	38 year old male	Case study		SL buprenorphine	Koro (acute anxiety due to the perception of intraabdominal penile retraction/shrinkage and fear of impending death)	1
Chua [105]	Singapore, Singapore	Mid-20 s male and mid-30 s female	Case series		SL buprenorphine	Cellulitis	1
DelGiudice [106]	Fréjus, France	13 clients aged 25–43	Case series	1996–2001	SL buprenorphine	Injection-site abscesses, cellulitis in multiple sites, thrombophlebitis in multiple sites, cutaneous necrosis	2
Eiden a [107]	Montpellier, France	31 clients (median age: 39)	Quantitative descriptive	2012–2013		Skin and soft-tissue infection, sepsis, endocarditis, spondylitis, meningitis, pulmonary abscess, candidemia	2
Eiden b [108]	Montpellier, France	192 clients (median age: 34)	Quantitative descriptive	2002–2012	SL buprenorphine	Cutaneous abscesses, osteoarticular infections, pulmonary infections, venous infections, endocarditis, hepatitis, sepsis, puffy hand syndrome	3
Eiden [109]	Languedoc-Roussillon region, France	198 people with extramedical use of buprenorphine	Quantitative descriptive	2002–2012	SL buprenorphine	Cutaneous abscess, venous infection, puffy hand syndrome, osteoarticular infections, endocarditis, pulmonary infections, sepsis, hepatitis	5
Espitia [110]	Nantes, France	33 year old male	Case study			Nicolau Syndrome (necrotic-centre lesion with nerve ischemia and motor deficiency)	1
Feeney [75]	Brisbane, Australia	24 year old female	Case study		SL buprenorphine	Femoral abscess with groin tissue necrosis	1
Gautschi [111]	Perth, Australia	30 year old male	Case study			Groin tissue abscess and myositis	1
Hakkinen [112]	Finland	225 postmortem toxicology cases with a urine sample positive for buprenorphine, norbuprenorphine or naloxone and background information supporting drug use	Quantitative descriptive	2010–2011		Fatal overdose	3
Ho [113]	Singapore, Singapore	131 people who use IV buprenorphine	Quantitative descriptive	2004–2006	SL buprenorphine	Cellulitis, abscess, necrotizing fasciitis, false aneurysms, thrombophlebitis, hematoma, lymphadenopathy, infection of specific site	4
Horyniak [76]	Melbourne, Australia	23 people who use IV buprenorphine	Mixed methods	2006	SL buprenorphine/BNX (mixed with saliva or lemon juice)	Nausea, vomiting, abscesses, vein damage	4
Jenkinson [62]	Melbourne, Australia	156 PWID	Quantitative descriptive	2002	SL buprenorphine	Overdose, abscesses/infections, scarring/bruising, thrombosis	4
Joethy [114]	Singapore, Singapore	29 year old male	Case study		SL buprenorphine	Mechanical nerve injury	1
Kintz [115]	Multiple sites in France	38 and 79 fatalities involving buprenorphine	Quantitative descriptive	1996–2000	SL buprenorphine	Fatal overdose	3
Kintz [116]	Strasbourg, France	13 fatalities involving buprenorphine	Quantitative descriptive	2000–2001	SL buprenorphine	Fatal overdose	2
Kluger [117]	Montpellier, France	31 year old male	Case study		SL buprenorphine	Penile and scrotal skin necrosis	1
Kriikku [63]	Finland	775 toxicology cases positive for buprenorphine upon death	Quantitative descriptive	2010–2014	SL buprenorphine/BNX	Overdose	5
Kulaksızoglu [77]	Antalya, Turkey	19 year old male	Case study	2018	SL BNX (dissolved in hot water)	Depressive symptoms	1
Kumar [50]	Madras, India	100 PWIDs	Quantitative descriptive	1998	SL buprenorphine	HCV, HIV, Hepatitis B	4
Lee [79]		mid-40 s female	Case study		SL buprenorphine (dissolved in hot water)	Blurred vision, bacterial endocarditis, mild cognitive impairment	1
Lee [118]	Singapore, Singapore	25 year old male	Case study		SL buprenorphine	Endocarditis, protein-losing enteropathy, tricuspid regurgitation, and heart failure	1
Lim [119]	Singapore, Singapore	7 males aged 28–53	Case series		SL buprenorphine	Loss of consciousness, left/right hemispheric syndrome (including hemianopia, gaze deviation, hemineglect, and aphasia), head injury, thrombophlebitis, arm cellulitis	2
Liu [80]	Multiple sites in China	1235 people with OUD and a history of buprenorphine use for at least three days	Quantitative descriptive	2000–2001	SL buprenorphine	Opioid withdrawal	4
Lo [44]	Singapore, Singapore	53 people with extramedical use of buprenorphine (mean age 34.5)	Case series	2005	SL buprenorphine	Skin infections; limb abscesses, ischaemia, and gangrene; necrotising fasciitis; septic arthritis; pseudoaneurysm of the femoral artery; infective endocarditis; withdrawal symptoms, hepatitis C; syncope/seizure; atypical chest pain; pulmonary tuberculosis	2
Loo [120]	Singapore, Singapore	4 males aged 22–55	Case series	2005	SL buprenorphine	Large thenar abscess, ischemic hand, subclavian vein thrombosis, sepsis, wet gangrene of the digits, paralysis of thenar muscles, carpal tunnel, amputation	2
Marka [121]	New Hampshire, USA	29 year old female and 37 year old male	Case series		SL BNX	Crospovidone reactions (skin foreign body reaction)	1
Nielsen [122]	Melbourne, Australia	250 people who had experience with OAT	Quantitative descriptive	2004–2005	SL buprenorphine	Overdose	3
Ojha [123]	Kathmandu, Nepal	300 PWIDs	Quantitative descriptive		SL buprenorphine	HIV infection	4
Partanen [124]	Helsinki, Finland	25 PWIDs ages 20–39	Case series	2000–2005	SL buprenorphine	Acute limb ischemia, limb infection (osteomyelitis), and amputation	2
Peyrière [60]	Montpellier, France	33 year old male and 50 year old male	Case series	2007	SL buprenorphine	Exacerbation of chronic HCV	2
Power [36]	Sydney, Australia	15,832 individuals who use IV buprenorphine	Quantitative descriptive	2001–2016	SL buprenorphine/BNX	Overdose	5
Prosser [125]	Sydney, Australia	32 year old male	Case study		Buprenorphine transdermal patch	Tubulo-interstitial nephritis	1
Puolakka [126]	Finland	6 men aged 16–21	Case series		SL buprenorphine	Cervical myelopathy and neck muscle rhabdomyolysis	1
Reynaud [127]	Alsace and Auvergne, France	6 cases of fatalities involving consumption of buprenorphine-benzodiazepine combinations	Case series	1996–1997	SL buprenorphine	Fatal overdose	1
Robinson [128]	Wellington, New Zealand	2 cohorts (54 and 44 people respectively) presenting for OUD treatment	Quantitative descriptive	1990–1992	SL buprenorphine/ BNX	Opioid withdrawal	3
Roux [47]	Multiple sites in France	111 clients receiving OAT with buprenorphine	Quantitative descriptive	2004–2005	SL buprenorphine	Overdose	5
Roux [57]	Multiple sites in France	371 PWID with OUD	Quantitative descriptive	2015	SL buprenorphine	Hand swelling, vein obstruction, rolling veins, cotton fever, and cutaneous abscesses	4
Seet^%^ [59]	Singapore, Singapore	18 year old male	Case study		SL buprenorphine (dissolved in water)	Diffuse cystic leukoencephalopathy	1
Seet^%^ [129]	Singapore, Singapore	27 year old male and 31 year old male	Case series		SL buprenorphine (dissolved in hot water)	Sciatic neuropathy, severe myositis, and rhabdomyositis	1
Seet^%^ [130]	Singapore, Singapore	51 people who use IV buprenorphine	Case series	2002–2005	SL buprenorphine	Cellulitis, endocarditis, myositis, abscesses; withdrawal, seizures, limb ischemia, respiratory failure, rhabdomyolysis, thrombosis, renal failure, leukoencephalopathy	3
Sharma [131]	Singapore, Singapore	32 year old male	Case study		SL buprenorphine (dissolved in hot water)	Myofasciitis (thighs) and polyneuritis	1
Singh [86]	India	24 year old male	Case study		Buprenorphine ampoules	Generalized tonic–clonic seizures; withdrawal; premature ejaculation	1
Singh [87]	Chandigarh, India	18 people with extramedical buprenorphine use	Case series	1987–1990	Buprenorphine ampoules	Concurrent use of benzodiazepines; pain, insomnia, nasal symptoms, irritability, restlessness, muscle twitching, diarrhea, palpitations (withdrawal); gastric antral erosions	3
Tan [132]	Singapore, Singapore	15 patients aged 25–58 who underwent surgery for pseudoaneurysms due to chronic injection drug use	Case series	2005–2008	SL buprenorphine	Infected pseudoaneurysm; gangrene; wound infection; rebleeding	3
Teo [133]	Singapore, Singapore	49 year old male	Case study		SL buprenorphine	Tetanus	1
Vicknasingam [90]	Multiple sites in Malaysia	276 people who use IV buprenorphine; for the second wave 77/276 were re-interviewed 77/276 and additional 171 new participants included	Mixed methods	2006–2007	SL buprenorphine for first survey, BNX in second survey	Weight loss, muscle fatigue, difficulty breathing, chest discomfort	3
Victorri-Vigneau [134]	Nantes, France	16 people who use IV buprenorphine	Case series		SL buprenorphine	Pain, burning, necrosis on injection site; thrombosis/livedo	2
White [91]	Multiple sites in Australia	16 people who inject BNX films	Qualitative	2012–2013	Buccal BNX (films; spitbacks)	Injection site problems; puffy hands; perceived heart disturbance; opioid withdrawal	5
Wilson [135]	Kentucky, USA	10 clients evaluated for ischemia of the hand or digits after injection of BNX	Case series	2011–2015	SL BNX	Hand ischemia; dry gangrene	2
Winslow [92]	Singapore, Singapore	120 people with extramedical use of buprenorphine, enrolled in treatment	Quantitative descriptive	2005–2006	SL buprenorphine	Skin and soft tissue infections, gangrene, thrombophlebitis, acute hepatitis, septicaemia with endocarditis, multiple lung abscesses, opioid withdrawal, embedded foreign body removal	3
Winslow [93]	Singapore, Singapore	106 PWIDs presenting to an addictions management programme	Quantitative descriptive	2005–2006	SL buprenorphine	HCV	5
Yang [136]	Singapore, Singapore	48 year old M and 30 year old female	Case series		SL buprenorphine	Septic sacroiliitis (progressive back pain, limitation of movement, fever)	1
Yeo [95]	Singapore, Singapore	8 clients aged 26–46	Case series	2005	SL buprenorphine (tablets; dissolved in hot water)	Arterial pseudoaneurysm, infective venous thrombus, venous thrombus, end arterial spasms, and sympathetic dystrophy; amputation of lower limb	2

*BNX* buprenorphine/naloxone, *HCV* Hepatitis C virus, *HIV* Human Immunodeficiency Virus, *IV* intravenous, *OAT* opioid agonist therapy, *OUD* opioid use disorder, *PWID* people who inject drugs, *SL* sublingual

% = Seet (2007) appears to contain data that is also presented in Seet (2005) and Seet (2006)

After reviewing the included studies, we chose to group them as studies primarily reporting on dose and frequency of use among regular buprenorphine injectors in Table 1 and those reporting on adverse events in Table 2. Where studies reported on both of those outcomes, they are included in both tables.

Studies included in Table 1, that is, those reporting on persons who regularly inject buprenorphine were of diverse design, but largely quantitative descriptive studies (surveys or incidence/prevalence studies without a comparison group) and were published between 1984 and 2018. Both oral buprenorphine-alone and BNX formulations were reportedly injected, and in countries in which the liquid formulation is available (Iran, India for example), injection of ampoules was also described. There was heterogeneity related to the frequency of injection among regular buprenorphine users. Our results revealed a report of injecting a maximum of 10 times daily however more common were reports of injecting 2–4 times daily. Among studies reporting injection of buprenorphine ampoules, doses ranged from < 1 mg/day to 24 mg/day. Among those studies reporting on the injection of oral buprenorphine or BNX, doses reported were between < 1 mg to 12 mg daily. Sixty-seven percent of (28/42) studies included in Table 1 had MMAT scores ≤ 3 indicating low quality and high risk of bias. Many were limited by selection bias and measurement bias.

Adverse events associated with buprenorphine injection are reported in Table 2. Adverse events described were generally either known side effects associated with opioids/buprenorphine (overdose, precipitated withdrawal), injection-related complications (endocarditis, cellulitis etc.) or theorized to be as a result of excipients in the buprenorphine/BNX tablets [44]. Adverse events were associated with injection of oral formulations of buprenorphine/BNX although one case report described adverse events associated with injection of buprenorphine from a transdermal patch. The quality of included studies is presented in Table 2. Most studies (53/67, 79%) were of low quality based on MMAT scores ≤ 3 and were judged to be at high risk bias.

## Discussion

Although existing literature has synthesized and described the extent and motivations for extramedical buprenorphine use [33, 45, 46], our review is the first to systematically document patterns of injection and adverse events among people who inject buprenorphine regularly. The studies summarized here could be characterized as coming from countries where either diverted oral buprenorphine is easily accessible (i.e. France [47, 48], Singapore [49]), or, from countries in which more desirable opioids (ie. heroin) are difficult, expensive, or dangerous to obtain (ie. India [50], Bangladesh [51], Finland [52, 53]).

Whereas most people who use buprenorphine extramedically do so irregularly and to manage or mitigate opioid cravings or withdrawal [33, 45, 54–56], our findings demonstrate that there is a smaller subset of persons who inject extramedical buprenorphine for its reinforcing properties. The use of buprenorphine in this way across multiple jurisdictions suggests that BUP iOAT may be an acceptable treatment option for persons with severe, refractory OUD that is non responsive to traditional OAT, or who are not interested in OAT. Possible acceptability of BUP iOAT is further supported by a recent cross sectional study from France among 353 persons with treatment-refractory OUD, 83% of whom indicated they would be willing to consider BUP iOAT were it available [57]. Factors positively associated with willingness to receive BUP iOAT included a history of > 5 injection-related complications, history of regular buprenorphine injection (compared with heroin and prescription opioids), and no lifetime overdose [57]. Among those willing to receive BUP iOAT, willingness to receive supervised dosing was positively associated with injecting heroin, older age, and not having stable housing [57].

Our review documented daily doses of injected SL buprenorphine between < 1 mg-12 mg daily, which is less than the oral buprenorphine doses that best retain persons with OUD in treatment (> 16 mg [58]), likely reflecting higher effective doses when injected. These doses suggest that a BUP iOAT program may be feasible with the existing formulations of buprenorphine and would not require crushing and injecting large volumes of tablets or liquid. It should be noted however that most of these studies occurred in the pre-fentanyl era and therefore required doses for BUP iOAT would likely be higher among fentanyl-dependent persons.

The frequency of use reported among regular buprenorphine injectors (many reporting 2–4 times daily) is similar to the range in frequency of heroin typically injected. Taken together, the dosing and frequency of use of injected buprenorphine revealed in this review provide a starting place for possible dosing were a pilot BUP iOAT clinical trial established.

The adverse events documented as associated with injection of buprenorphine were largely known side effects associated with opioids/buprenorphine (overdose, precipitated withdrawal), injection-related complications (endocarditis, cellulitis etc.), or theorized to be as a result of excipients in the buprenorphine/ BNX tablets [44, 59]. Reports of acute hepatitis in the context of chronic hepatitis C are worth noting [60, 61]. Although overdose was reported, it was most-commonly reported in the context of concurrent sedative use [62, 63]. Consistent with buprenorphine’s known ceiling effect with respect to respiratory depression, observational studies suggest that extramedical use of buprenorphine is actually protective for overdose—data considering incidence of overdose following buprenorphine injection at a supervised consumption facility in Australia has demonstrated a significant protective effect associated with injecting buprenorphine compared with injection of heroin or other opioids [36]. Similarly, a recent study in Ohio, US found that higher frequency of extramedical buprenorphine use among people with OUD was associated with a lower risk of drug overdose [64]. Lower likelihood of overdose is a possible major benefit of a BUP iOAT intervention both for participants as well as for the broader community given concerns [65] that diverted doses could end up in the hands of children or other persons without opioid tolerance. However, properly powered trials would be needed to ensure the safety of such an intervention compared with usual treatment. With respect to the infectious complications reported, these are known injection-related complications and could be minimized with harm reduction education and provision of harm reduction supplies. Interestingly, precipitated withdrawal was not a commonly reported adverse-event. This may reflect publication bias, or alternately may reflect proficiency in timing other opioid use among regular buprenorphine injectors. As with SL buprenorphine induction, novel induction methods such as micro-dosing and macro-dosing may be useful for a potential BUP iOAT intervention in order to avoid precipitated withdrawal and retain persons who use fentanyl in treatment [66].

There are several limitations to this systematic review. Firstly, we included only English language publications, and did not search the grey literature, therefore, our review may have missed some relevant publications. Secondly, owing to the heterogeneity of the results, no attempt at meta-analysis was made. The studies included were generally of low quality. Individual studies were subject to selection bias, measurement bias and overall the dataset is subject to outcome reporting bias and language bias. Finally, the majority of the publications included are from outside of North America, and many were published decades ago. It is therefore unclear whether the findings would be applicable to persons with opioid use disorder in North America, and in the current context of a fentanyl-dominated drug supply.

Given the ongoing opioid crisis and increasingly toxic drug supply, there remains an urgent need for novel therapies for the treatment of OUD among persons not responsive to traditional therapies and/or among those not interested in them. Our results paint a clearer picture of the patterns of use of buprenorphine among real-world populations who regularly inject the drug, and could inform the development of a BUP iOAT intervention. Our results suggest that a BUP iOAT intervention could be safe and feasible. Importantly, although people who inject drugs in France demonstrated strong willingness to consider this type of therapy, it remains unclear if it would be acceptable to persons with OUD in the US or Canada. Take-home doses, and availability of liquid formulations may increase the acceptability of BUP iOAT among people with OUD in North America. Future research should work with persons with lived experience to explore acceptability, and consider testing the feasibility, efficacy and safety of BUP iOAT compared with traditional OAT and iOAT.

## Supplementary Information

**Additional file 1**. **Appendix 1**. Search strategy.

## Data Availability

All data generated or analysed during this study are included in this published article and its supplementary information files.
