# Correction: Patterns of use and adverse events reported among persons who regularly inject buprenorphine: a systematic review

**DOI:** 10.1186/s12954-022-00705-6

**Published:** 2022-11-07

**Authors:** Nikki Bozinoff, Vitor Soares Tardelli, Dafna Sara Rubin-Kahana, Bernard Le Foll

**Affiliations:** 1 Campbell Family Mental Health Research Institute, Centre for Addiction and Mental Health, Toronto, ON Canada; 2 Department of Family and Community Medicine, University of Toronto, Toronto, ON Canada; 3 Addictions Division, Centre for Addiction and Mental Health, Toronto, ON Canada; 4 Departamento de Psiquiatria, Universidade Federal de Sao Paulo, São Paulo, Brazil; 5 Translational Addiction Research Laboratory, Centre for Addiction and Mental Health, Toronto, ON Canada; 6 Department of Psychiatry, University of Toronto, Toronto, ON Canada; 7 Child, Youth, and Family Services, Centre for Addiction and Mental Health, Toronto, ON Canada; 8 Division of Neurosciences and Clinical Translation, Department of Psychiatry, University of Toronto, Toronto, ON Canada; 9 Department of Pharmacology and Toxicology, University of Toronto, Toronto, ON Canada; 10 Waypoint Research Institute, Waypoint Centre for Mental Health Care, Penetanguishene, ON Canada

## Correction to: Harm Reduction Journal (2022) 19:113 10.1186/s12954-022-00695-5

Following publication of the original article [1], the co-author would like to insert his middle name and it should read as “Vitor Soares Tardelli” rather than “Vitor Tardelli”.

The original article has been corrected.
