# Supplementary material for: Patterns of use and adverse events reported among persons who regularly inject buprenorphine: a systematic review
Source: Harm Reduct J. 2022 Oct 13;19:113. doi: 10.1186/s12954-022-00695-5 (PMC9559254; doi:10.1186/s12954-022-00695-5)
Supplement: Supplementary file 1 — Additional file 1. Appendix 1. Search strategy. [file 12954_2022_695_MOESM1_ESM.docx]

Appendix 1 - Search strategy

Databases searched: Ovid MEDLINE, Embase Classic, and APA PsycInfo

1. buprenorphine.af.

2. injection.af.

3. intravenous.af.

4. misuse.af.

5. abuse.af.

6. self-administration.af.

7. non-prescribed.af.

8. 2 or 3

9. 4 or 5 or 6 or 7

10. 1 and 8 and 9
